# Green MIP-Based Electrochemical Sensing Platform for Environmental Ivermectin Analysis

**DOI:** 10.1021/acsomega.6c00641

**Published:** 2026-04-30

**Authors:** Zeynep Aydemir, Beril S. Kaya, Setareh Dorreh, Abdullah Al Faysal, Taner Erdoğan, Ayşegül Gölcü

**Affiliations:** † Istanbul Technical University, Faculty of Sciences and Letters, Department of Chemistry, Maslak, 34467 Istanbul, Turkey; ‡ Istanbul Health and Technology University, Faculty of Pharmacy, 34445 Istanbul, Turkey; § Department of Chemistry, Faculty of Science, Atatürk University, Erzurum, 25240, Turkey; ∥ Kocaeli University, Kocaeli Vocational School, Department of Chemistry and Chemical Processing Technologies, 41380 Kocaeli, Turkey

## Abstract

Ivermectin (IVM), a macrocyclic lactone derived from *Streptomyces avermitilis*, is widely recognized as a “wonder drug” for its broad-spectrum efficacy against internal and external parasites in human and veterinary medicine. Owing to its potent pharmacological activity, precise quantification of IVM is essential for therapeutic monitoring and dose optimization. In this study, we report the design of a novel electrochemical sensor based on molecularly imprinted polymer (MIP) technology, specifically tailored for the selective detection of IVM. The sensor was fabricated via an electropolymerization strategy employing methacrylic acid (MAA) as the functional monomer and aniline as the comonomer in phosphate-buffered saline (PBS, pH 7.0). To the best of our knowledge, this represents the first electropolymerization-based MIP sensor developed for IVM determination. The resulting MAA-IVM@MIP/GCE sensor was thoroughly characterized using cyclic voltammetry (CV), electrochemical impedance spectroscopy (EIS), Fourier-transform infrared spectroscopy (FTIR), and scanning electron microscopy (SEM). Electrochemical detection was achieved through an indirect redox-probe approach with 5.0 mM [Fe­(CN)_6_]^3–^/^4–^, providing a wide linear range (1 × 10^–12^ −1 × 10^–11^ M) and remarkably low limits of detection (LOD: 2.91 × 10^–13^ M) and quantification (LOQ: 9.71 × 10^–13^ M). The sensor demonstrated high sensitivity, reproducibility, and selectivity, clearly distinguishing IVM from structurally related compounds. It maintained strong analytical performance in pharmaceutical formulations, biological matrices, and environmental samples such as tap water and soil, showing minimal matrix interference. These results confirm the platform’s robustness and applicability. Density functional theory (DFT) calculations were performed to evaluate template–monomer interactions and determine the optimal template:monomer ratio for the MIP-based sensor. The results revealed that the 1:1 complex exhibited the most favorable binding characteristics, consistent with the experimental findings. In addition, the sensor fabrication strategy was designed in accordance with green analytical chemistry principles. The electropolymerization process was performed in aqueous phosphate-buffered saline under mild conditions without the use of excessive cross-linkers or hazardous reagents. The approach minimizes organic solvent consumption, reduces energy requirements, and enables sensor reusability, thereby contributing to a sustainable and environmentally responsible analytical platform. Overall, this cost-effective, scalable, and environmentally conscious electrochemical sensor provides a practical tool for reliable IVM monitoring and has strong potential for clinical diagnostics, pharmacokinetics, and pharmaceutical quality control.

## Graphical abstract

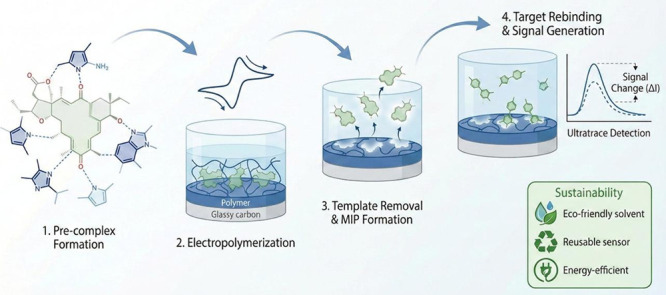

## 1 Introduction

Originally isolated from the actinomycete *Streptomyces avermitilis*, ivermectin (IVM) belongs to the macrocyclic lactone family of compounds.^1^ It marked a breakthrough in veterinary medicine as the first drug of its kind to be used against parasitic infections, and has since become the most extensively used agent for treating both internal and external parasites in animals.^2^ Beyond its antiparasitic properties, IVM has also been described as a “wonder drug” due to its broad range of biological activities, including antimicrobial, antiviral, and anticancer effects.^3^ Notably, its antiviral action extends to both DNA and RNA viruses. IVM inhibits nuclear transport by targeting the importin α/β1 heterodimer, which is essential for the translocation of viral proteins into the host cell nucleus, thereby disrupting viral replication processes.^4^ Given its broad biological profile, IVM was even investigated during the COVID-19 pandemic as a potential treatment option. Although later studies yielded inconclusive results, their widespread use during that period highlights the interest in repurposing established drugs during global health emergencies.^5^

As IVM exhibits multiple biological effects, ensuring accurate methods for its detection and quantification has become a key focus in both scientific and medical fields. Over the years, various detection techniques have been developed and refined to meet this need. Early detection approaches employed thin-layer chromatography (TLC), enabling qualitative identification of IVM as a fluorescent derivative in cattle dung, with a limit of detection (LOD) as low as ≤40 ng/g.^6^ Subsequent advancements led to competitive enzyme immunoassays developed for bovine liver samples, offering a LOD as low as 1.6 ng/g and demonstrating strong correlation with high-performance liquid chromatography (HPLC) results.^7^ For food safety monitoring, immunoaffinity column cleanup combined with reversed-phase HPLC allowed IVM quantification in swine liver with a LOD of 2 μg/kg and recovery rates ranging from 85% to 102%.^8^ In aquaculture, an HPLC method with fluorescence detection enabled precise measurement of IVM residues in Atlantic salmon tissues, with a sensitivity of 1 ng/g, liver being the tissue with the highest accumulation.^9^ Recent efforts have focused on immunochemical methods: a monoclonal antibody-based indirect competitive enzyme-linked immunosorbent assay (ic-ELISA) achieved a LOD of 0.77 ng/mL, while a lateral-flow immunochromatographic assay (ICA) provided a visual detection limit of 25 ng/mL and a scanner-based LOD of 2.9 ng/mL.^10^ These methods provide accurate and sensitive results, but their application is restricted due to the high cost, slow processing, and requirement for expert handling and complex tools.

Compared to the aforementioned techniques, electrochemical methods offer several advantages, including lower limits of detection, rapid analysis time, cost-effectiveness, and suitability for miniaturization.^11^ These features make them particularly attractive for sensitive, on-site, and real-time monitoring of IVM residues in various matrices. Furthermore, employing surface modification techniques can significantly improve the electrochemical performance of working electrodes, enhancing both sensitivity and selectivity. One promising approach involves the use of molecularly imprinted polymers (MIPs), which are cost-effective, durable, and demonstrate excellent performance and stability under a range of conditions.^12^ Thanks to their mechanical strength, thermal stability, and ease of transport, MIPs have gained significant interest as analytical tools in pharmaceutical applications. These polymers can be synthesized with minimal expense from readily available materials, offering notable sensitivity that supports their integration into a variety of applications—most notably, electrochemical sensor technologies.^13^ To fabricate an MIP on the surface of an electrode, functional monomers are combined with a target template and a cross-linking agent to form a structured polymer matrix. Once polymerization is complete, the template is removed, leaving behind specific binding sites that are complementary in shape, size, and chemical functionality to the original molecule.^14,15^ The rigidity of the polymer matrix is also recognized as a key parameter governing MIP performance, as higher structural stiffness generally improves the stability and fidelity of the imprinted cavities, enhancing selectivity, while overly rigid matrices may limit analyte diffusion and binding kinetics. This balance between mechanical stability and accessibility has been highlighted as an important design consideration in molecular imprinting.^16^ In recent years, computational approaches have become valuable tools in the design of MIPs, particularly for predicting interactions between monomers and templates in the prepolymerization phase.^17^ Among these methods, density functional theory (DFT) has proven especially useful in identifying optimal functional monomers based on calculated interaction energies with the target template. DFT has been effectively employed in selecting both monomers and solvents for MIP synthesis using templates.^18^

In recent years, the development of analytical methods has increasingly been guided by the principles of green analytical chemistry (GAC), which aim to minimize environmental impact while maintaining high analytical performance.^19^ Key green profile parameters include the reduction of hazardous reagents, minimization of organic solvent consumption, lower energy requirements, and the design of reusable and waste-reducing analytical platforms. Electrochemical sensing technologies, particularly those based on MIPs, align well with these principles due to their low reagent consumption, mild operating conditions, and potential for miniaturization and reuse. Therefore, integrating green chemistry considerations into sensor design not only enhances environmental sustainability but also improves the practicality and scalability of analytical methods, and such aspects can be systematically evaluated using modern greenness assessment metrics.^20^

Despite the significant progress achieved with chromatographic, immunochemical, and spectroscopic techniques for IVM determination, many of these approaches still rely on expensive instrumentation, labor-intensive sample preparation, and centralized laboratory settings, which limit their suitability for rapid and on-site monitoring. Electrochemical sensing platforms, particularly those incorporating MIPs, have emerged as technologically relevant alternatives due to their high sensitivity, portability, and cost-effectiveness. In this context, integrating computationally guided MIP design with electrochemical transduction represents a promising strategy to enhance selectivity while maintaining operational simplicity. Therefore, the development of new MIP-based electrochemical sensors tailored for IVM detection remains highly relevant within the current landscape of analytical technologies.

In this study, we developed and optimized a novel electrochemical sensing platform by combining MIP technology with electrochemical techniques for the selective and sensitive detection of IVM. The polymer matrix was prepared using methacrylic acid (MAA) as the functional monomer and aniline as the comonomer in a phosphate-buffered saline (PBS, pH 7.0) solution. The electro polymerization was carried out directly on the surface of a glassy carbon electrode (GCE) using cyclic voltammetry, yielding a stable, imprinted polymeric film.

The morphological and electrochemical properties of the synthesized MIP were characterized using scanning electron microscopy (SEM), Fourier-transform infrared spectroscopy (FTIR), electrochemical impedance spectroscopy (EIS), and cyclic voltammetry (CV). The resulting MAA-IVM@MIP/GCE exhibited strong molecular recognition capability toward IVM, even in the presence of structurally similar compounds. Compared to previously reported sensors, the developed platform showed enhanced sensitivity and selectivity. Moreover, its successful application to pharmaceutical and environmental samples highlights its practical applicability. Overall, this electrochemical sensor provides a reliable, efficient tool for accurate detection of IVM, contributing considerably to drug monitoring and quality control efforts in the pharmaceutical and veterinary fields.

## 2 Experimental Section

### 2.1 Reagents and Chemicals

In this study, IVM was obtained from Istanbul University, Drug Research Center, and the tablet form was obtained upon request from Deva HoldingAdditional chemicals were sourced from Sigma-Aldrich (Darmstadt, Germany), including methacrylic acid (MAA, ≥99.0%), aniline (≥99.5%), sodium phosphate monobasic (≥99.0%), sodium phosphate dibasic (≥99.0%), dopamine (DOP, 99.0%), sodium sulfate (Na_2_SO_4_, >97.0%), uric acid (UA, ≥99.0%), potassium nitrate (KNO_3_, ≥ 99.0%), acetonitrile (ACN, 99.9%), ascorbic acid (AA, ≥99.0%), potassium ferrocyanide and ferricyanide (≥99.0%), paracetamol (PAR, ≥99.0%), acetone (99.5%), magnesium chloride (MgCl_2_, ≥98.0%), potassium chloride (KCl, ≥99.0%), 2-hydroxy-2-methylpropiophenone (≥97.0%), acetic acid (HAc, 99.0%), methanol (MeOH, 99.9%), ethanol (EtOH, 99.5%), hydrochloric acid (37%), and sodium hydroxide (>97%). All substances were employed without any further purification or processing.

Stock solutions were prepared as follows: IVM was dissolved in ethanol to obtain a 10 mM solution; MAA was dissolved in deionized water to a concentration of 10 mM; and an equimolar 5 mM solution of potassium ferrocyanide/ferricyanide was prepared in 0.1 mM KCl. Each solution underwent 10 min of sonication using an ultrasonic bath and was then stored at 4 °C until use. All preparations were conducted using ultrapure water maintained at 25 °C.

### 2.2 Equipment/Apparatus

Electrochemical measurements, including CV and differential pulse voltammetry (DPV), were conducted using a potentiostat/galvanostat (AUTOLAB PGSTAT302N, Metrohm, Utrecht, Netherlands) controlled through NOVA software version 2.1.6. EIS measurements were conducted using a PalmSens potentiostat (PalmSens BV, Houten, Netherlands) with PSTrace software version 5.12.1031. The electrochemical setup employed a conventional three-electrode system, consisting of an Ag/AgCl reference electrode (3 M KCl), a platinum wire serving as the counter electrode, and a glassy carbon electrode (GCE, 3.0 mm diameter) modified with either a MIP or a nonimprinted polymer (NIP) as the working electrode.

Reagents were accurately measured with an analytical balance (Ohaus Corporation, Shanghai, China). Drug extraction and rebinding processes were performed using a thermoshaker (Biosan TS-100, Riga, Latvia). Additional instruments used throughout the experimental procedures included an ultrasonic bath (JP Selecta, Barcelona, Spain) and a vortex mixer (ISOLAB Laborgeräte GmbH, Germany).

For morphological analysis of the polymer films, SEM was performed using a Tescan GAIA3 SEM-FIB system (FEI Quanta FEG 250, USA). To examine the chemical structure of the polymeric materials, ATR–FTIR spectroscopy was performed using a Shimadzu IRSpirit-T spectrometer (Shimadzu, Japan), spanning the mid-infrared range of 4000–500 cm^–1^.

### 2.3 Preparation Processes of EP-IVM@MIP/GCE and EP-IVM@NIP/GCE Sensors

Before electropolymerization, the GCE was cleaned by sonicating for 15 min in a 1:1 (v/v) solution of double-distilled water and methanol. The electrode surface was then polished with an alumina slurry, rinsed thoroughly with double-distilled water, and air-dried at room temperature.

To prepare the electropolymerization (EP) solution, a mixture containing 100 μL of IVM (10.0 mM), 100 μL of methacrylic acid (MAA, 10.0 mM), 500 μL of aniline (0.1 M), and 4300 μL of phosphate-buffered saline (PBS, pH 7.0) was used. A total of 5 mL of this solution was transferred into an electrochemical cell, where the GCE was immersed to carry out the EP process. Electropolymerization was performed by CV over 10 cycles within the potential window of −0.2 to +1.2 V at a scan rate of 50 mV/s. After polymer formation, the electrode was rinsed thoroughly with distilled water.

To remove the template molecule (IVM), the modified electrode was immersed in an EtOH:ACN mixture (2:1, v/v) and incubated on a thermoshaker at 25 °C and 650 rpm for 10 min. Subsequently, rebinding of IVM (5 × 10^–11^ M) to the vacant imprinted sites was achieved by incubating the electrode under the same thermoshaking conditions for 15 min.

Control experiments were conducted using NIP, which were synthesized under identical conditions but without the addition of IVM. The electrochemical performance of the sensors was evaluated using a 5.0 mM [Fe­(CN)_6_]^3–^/^4–^ redox probe by CV and DPV, and the results were compared with those obtained for the nonmolecular imprinted polymer (NIP)-modified GCE.

### 2.4 Analysis of IVM in Tablet Form and Serum Sample Applications

The practical performance of the fabricated sensor was evaluated using a commercial IVM tablet formulation. Five tablets were accurately weighed and crushed into a fine powder using a mortar. Based on the labeled content (3 mg IVM per tablet), an appropriate amount of the powdered sample was calculated and dissolved in ethanol to prepare a 1.0 mM IVM stock solution. This solution was then sonicated for 15 min to ensure complete dissolution. Serial dilutions were subsequently prepared from the stock solution to construct a calibration curve and conduct recovery studies, validating the sensor’s performance in detecting IVM within the pharmaceutical matrix.

For serum analysis, 0.5 mL of a 1 mM IVM solution was mixed with 2.7 mL of acetonitrile and 1.8 mL of drug-free human serum (previously stored at −20 °C) in a centrifuge tube. To remove proteins, the sample was centrifuged at 5000 rpm for 20 min. The resulting clear supernatant was carefully collected and diluted with ethanol for further calibration and recovery studies. All measurements were carried out in triplicate to assess the reproducibility and precision of the analytical method, and the relative standard deviation (RSD) was calculated for each data set.

### 2.5 Recovery Studies of IVM from Environmental Matrices

The aqueous stock solution was prepared by diluting 1 mL of a 1 mM drug solution with 9 mL of tap water to obtain a 0.1 mM mixture, which was then used for recovery tests at three concentration levels. Tap water samples were spiked with IVM at 1 × 10^–12^ M, 2.5 × 10^–12^ M, and 5 × 10^–12^ M, after which standard IVM solutions of 6.5 × 10^–12^ M, 5 × 10^–12^ M, and 2.5 × 10^–12^ M were added to the corresponding samples. The resulting data were used to calculate average recovery (%) and bias (%) to evaluate method accuracy.

Soil samples collected from the Istanbul Technical University campus were dried to homogenize and then heated to 105 °C to remove residual moisture. One gram of the dried soil was combined with 10 mL of deionized water, treated in an ultrasonic bath to enhance analyte release, and then centrifuged to separate the extract. One milliliter of the clear supernatant was diluted to 10 mL to produce the soil extract stock solution. Recovery assessments were performed using this extract by adding standard solutions (6.5 × 10^–12^ M, 5.0 × 10^–12^ M, and 2.5 × 10^–12^ M) to create three fortified levels. The sensor was used to analyze these samples, and the measured concentrations were compared to the expected values to determine recovery and bias. Recoveries between 95.0% and 105.0% were deemed satisfactory, confirming the method’s suitability for soil analysis.

### 2.6 Computational Studies

Density functional theory (DFT) calculations were performed to determine the optimum template:monomer ratio for the MIP-based sensor and to investigate possible template-monomer interactions. Initially, the three-dimensional structure of ivermectin was constructed, and a conformational analysis was carried out to identify the most stable conformer. Geometry optimization of this conformer was then performed using B3LYP (Becke, three-parameter, Lee–Yang–Parr hybrid functional)^21,22^ functional in combination with the 6–311G­(d,p) basis set. Based on the optimized geometry of ivermectin, template-monomer complexes were generated at template:monomer ratios ranging from 1:1 to 1:5. The initial geometries of these complexes were obtained using AutoDock Tools^23^ and AutoDock Vina.^24^ Subsequently, DFT calculations employing the same level of theory were performed to optimize the geometries of each complex and to calculate their corresponding energies (Δ*E*_*complex*_). In the next step, for each complex, the optimized geometric structure was first modified by removing the monomer unit(s) from the complex, yielding the template-only structure. Energy calculation was then performed at the same level of theory, and the corresponding energy values (Δ*E*_*template*_) were obtained. The same procedure was subsequently applied by removing the template molecule from the geometry-optimized complex, resulting in a structure composed solely of the monomer units. Again, energy calculation was carried out, and the corresponding energy values (Δ*E*_*monomer*(*s*)_) were determined. Finally, the binding energy for each complex was calculated by the following formula:

1 $${\Delta E}_{binding}={\Delta E}_{complex}-\left({\Delta E}_{template}+{\Delta E}_{monomer\left(s\right)}\right)$$

DFT calculations were performed with the use of Gaussian^25^ and GaussView^26^ software packages. VeraChem Vconf^27^ was used in conformational analysis and Discovery Studio Visualizer^28^ was used to visualize the results.

## 3 Results and Discussion

### 3.1 Surface Characterization

A detailed morphological analysis of the sensor surfaces was conducted using SEM, with the primary objective of investigating surface structural features and differentiating between the MIPs and NIPs. As depicted in Figures 1A-D, the SEM images provided clear evidence of a significant contrast between the MIP and NIP surfaces of the sensor. The MIP-modified surface exhibited a porous, rough morphology, consistent with the expected imprinting results (Figures 1A-C). These specific structural features indicate the successful formation of recognition cavities designed for the target analyte. The presence of porosity and surface irregularities is critical for enhancing the analyte’s binding capacity. Conversely, the NIP surface exhibited a notably smooth and uniform texture, lacking the porous characteristics of the MIPs, a result anticipated by the deliberate omission of template molecules during polymerization (Figure 1D). Ultimately, the SEM analysis confirmed the success of the imprinting process by visually demonstrating distinct morphological differences between the MIP and NIP surfaces.

**Figure 1:**
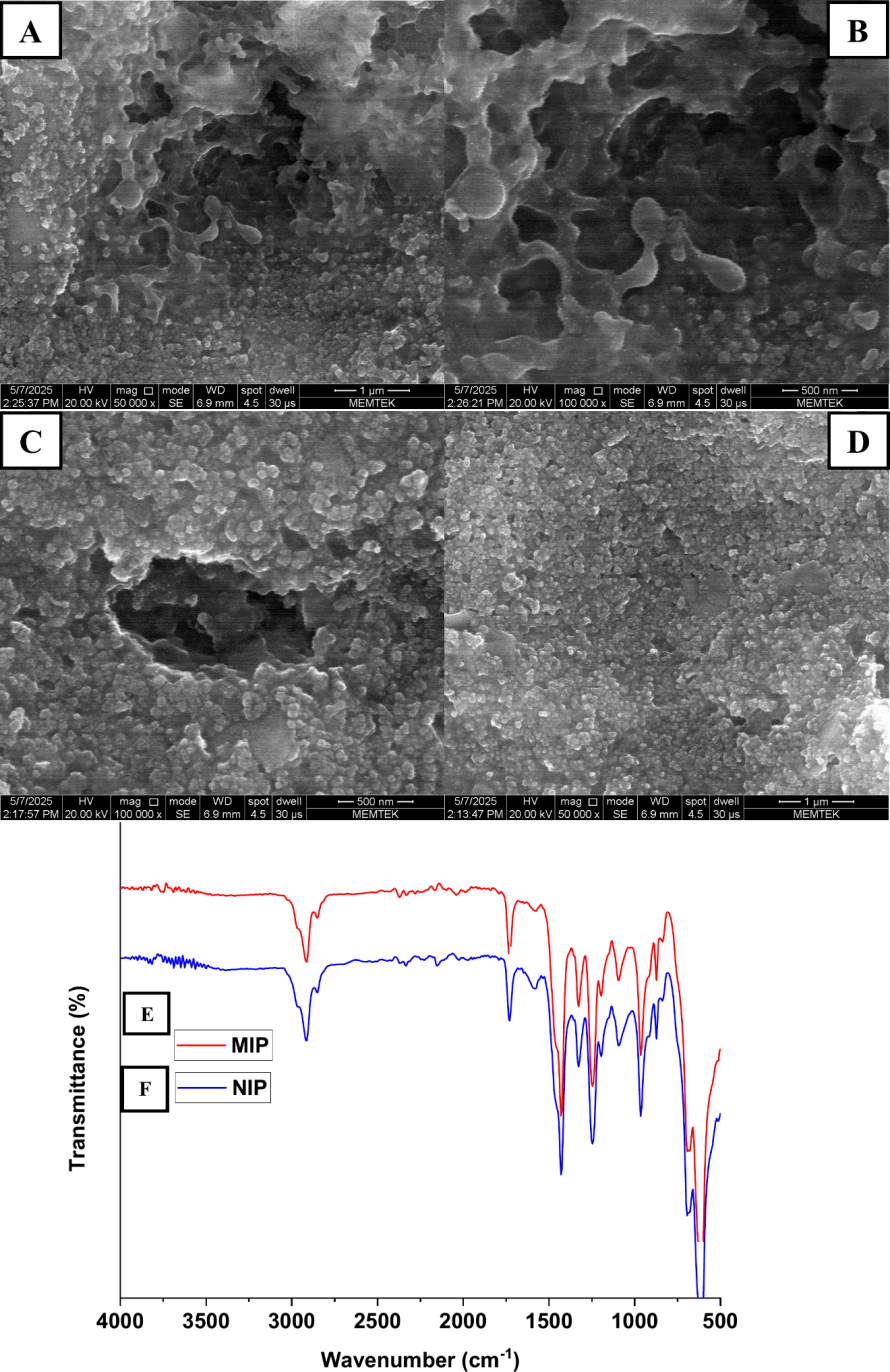
Surface characterization of the modified electrode. SEM micrographs showing the surface morphology of the MIP-modified electrode (A–C) and the NIP-modified electrode (D); ATR–FTIR spectra corresponding to the MIP (E) and NIP (F) films.

The functional groups of the chemical components employed in sensor fabrication were characterized by FTIR spectroscopy. Figures 1E and 1F present the FTIR spectra of both MIP (red line) and NIP (blue line) films. A broad absorption band observed in the range of 3000–3500 cm^–1^ is attributed to the stretching vibrations of N–H groups from aniline and the – OH groups from methacrylic acid. The C–H stretching vibrations of CH and CH_2_ groups appear between 2900 and 3000 cm^–1^. A distinct band around 1700 cm^–1^ is indicative of the C=O stretching vibration associated with methacrylic acid. Additionally, peaks related to the aromatic ring of aniline are observed in the fingerprint region (below 1600 cm^–1^), specifically around 1600 cm^–1^ and 1500 cm^–1^ due to C=C stretching vibrations of the aromatic ring, and C–N stretching vibrations around 1300 cm^–1^. The overall similarity in the FTIR spectra of MIP and NIP films suggests that the basic polymeric backbone formed from methacrylic acid and aniline is consistent between both formulations, with any differences due to the template (IVM) being subtle and not prominently visible as distinct new peaks, but rather potentially as slight shifts or intensity changes if significant interactions occur. These spectral features collectively verify the successful incorporation of functional groups into the polymeric matrix.

### 3.2 Electrochemical Characterization

The electrochemical response of the MAA-IVM@MIP/GCE sensor was assessed by CV at various fabrication stages—namely after polymerization, template extraction, and subsequent rebinding—using a 5 mM [Fe­(CN)_6_]^3–^/^4–^ redox couple in 0.1 M KCl. As depicted in Figure 2A, notable variations in anodic and cathodic peak currents were observed throughout the modification process. The bare electrode displayed the highest peak currents, owing to the unobstructed electron transfer at its surface. Following electropolymerization, a substantial reduction in peak currents was observed, indicating that the polymer layer impeded electron transfer and confirming successful imprinting. Removal of the IVM template led to the formation of recognition cavities within the polymer matrix, thereby increasing redox activity, as evidenced by higher peak currents than those of the polymer-coated sensor. Finally, upon rebinding of IVM at a defined concentration, a subsequent decrease in peak currents was detected, attributed to the occupation of the imprinted sites by IVM molecules, thus again limiting electron transfer at the MIP-modified electrode surface.

**Figure 2:**
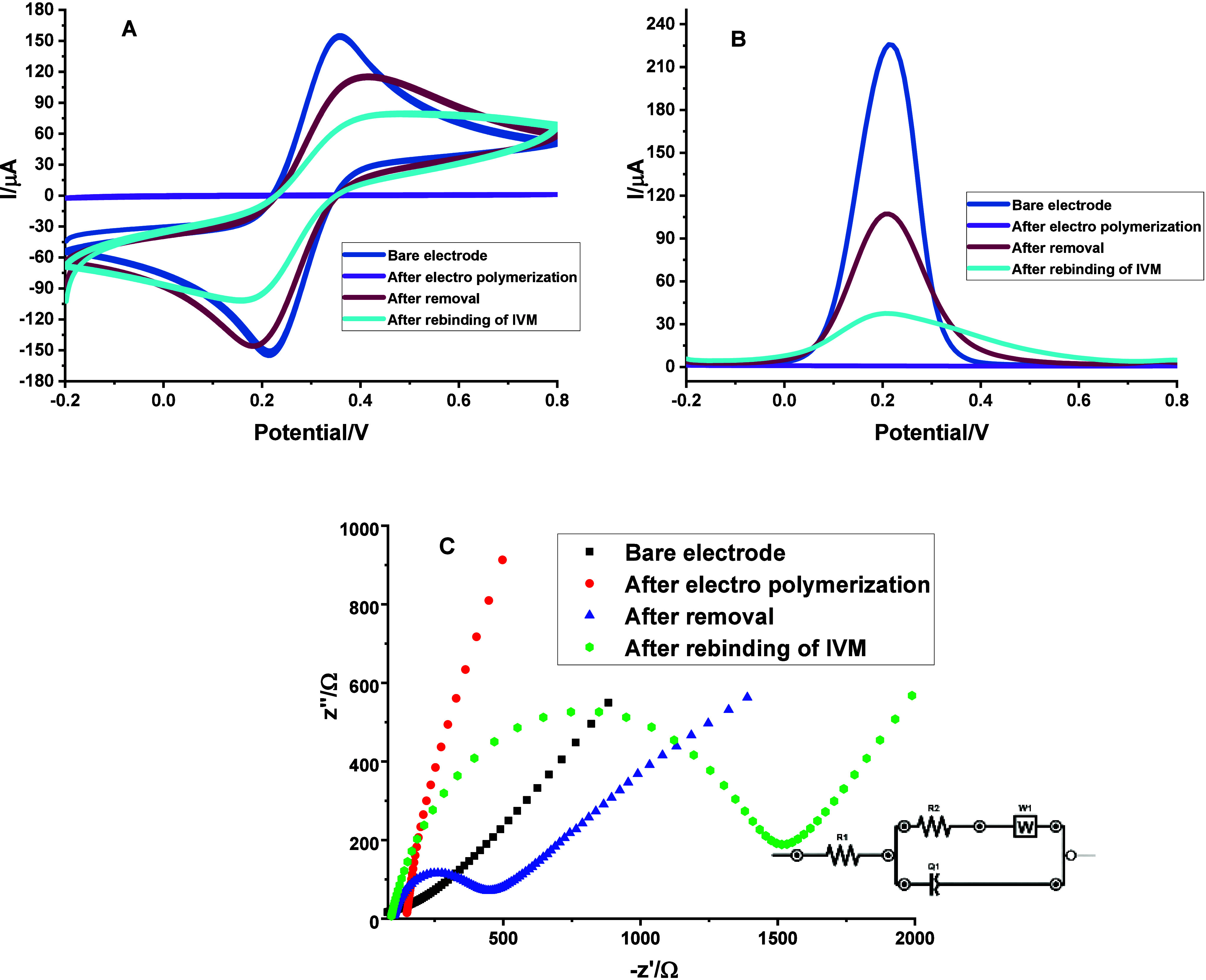
CV (A), DPV (B), and EIS (C) measurements of the GCE recorded at different stages: before polymerization, following polymer formation, after template removal, and after IVM rebinding, using a 5.0 mM [Fe­(CN)_6_]^3–^/^4–^ solution (for CV and DPV potential scan range: −0.2 to +0.8 V, scan rate: 0.05 V/s, and step potential: 0.01 V; for EIS, minimum frequency: 0.1 Hz, maximum frequency: 100,000 Hz, and *E*_ac_: 0.01 V).

Figure 2B depicts the DP voltammogram responses acquired from a standard analyte solution at different phases of the fabrication process of the MAA-IVM@MIP/GCE sensor. The peak current shows a reduction following the polymer formation, which is then succeeded by an increase after the template is removed, a phenomenon attributed to the creation of imprinted cavities. Following this, there is yet another decline in peak current during the analyte rebinding process, thus confirming the successful fabrication of the sensor and its specific recognition abilities.

The stepwise modification of the electrode was characterized using EIS to evaluate the interfacial charge-transfer resistance (R_ct_). As shown in the Nyquist plots (Figure 2C), the bare electrode displayed an initial R_ct_ of 155.6 Ω. Following electropolymerization, the deposition of the polymer coating caused a sharp increase in impedance to 4315 Ω. After the template removal step, the R_ct_ value decreased significantly to 656.5 Ω, reflecting improved conductivity through the newly created molecular footprints. Finally, the rebinding of IVM led to a renewed increase in impedance (R_ct_ = 1332 Ω), as the analyte filled the imprinted pores and restricted electron transfer.

### 3.3 Optimization Parameters

#### 3.3.1 Monomer-to-Template Ratio

The monomer-to-template ratio is a key factor in producing stable, efficient polymers, as it directly governs the bonding interactions between the functional monomer and the template. Choosing a suitable monomer helps reduce nonspecific interactions while promoting selective binding with the target analyte. In this work, MAA was employed as the functional monomer. To determine the optimal molar ratio, changes in peak current (ΔI_1_) before and after template extraction were examined at ratios ranging from 1:1 to 1:5, as shown in Figure S1A. The 1:1 ratio yielded the most favorable outcome, resulting in a robust polymer framework with distinct, selective cavities. At higher ratios, this led to heterogeneous monomer distribution, disrupting the formation of selective binding sites. Thus, the 1:1 ratio was identified as the most effective balance, ensuring efficient template removal and polymerization, as evidenced by the highest ΔI_1_ value.

#### 3.3.2 Number of Cycles for EP

The durability and effectiveness of the polymeric film are strongly influenced by the number of scans applied during the EP process. After selecting suitable monomers and their ratios, CV was performed for 3, 5, 7, 10, and 15 cycles to achieve an optimal thickness and stable polymer layer. The evaluation was based on changes in peak current values after template removal and EP. Beyond ten cycles, the peak current response began to decline **(** Figure S1B **)**. Consequently, 10 cycles were identified as the most reliable and efficient condition for EP.

#### 3.3.3 Removal Solution and Time

Template removal is a pivotal step in MIP preparation, as it ensures the formation of specific recognition sites. To determine the most suitable solvent, several candidates were tested, including acetone, acetonitrile, ethanol, methanol, glacial acetic acid (17.5 M), acetic acid (10 M), as well as binary mixtures of ethanol–acetonitrile (1:1, v/v) and methanol–acetonitrile (1:1, v/v). Among these, the ethanol–acetonitrile mixture exhibited the most excellent efficiency for template extraction, as reflected by the highest ΔI_1_ values (Figure S1C).

Following solvent optimization, the influence of removal time was evaluated using a thermoshaker over 3 to 15 min. The results indicated that ΔI_1_ peaked at 10 min **(** Figure S1D **)**. Extending the removal period beyond this point resulted in reduced performance, likely due to polymer chain degradation, partial pore collapse, and undesired recross-linking within the matrix. Hence, a removal time of 10 min was selected as the optimal condition for subsequent analyses.

#### 3.3.4 Rebinding Time

The rebinding step is a critical parameter that influences both the efficiency and the duration of the analysis. To determine the optimal rebinding time for stable, effective binding, variations in peak current (ΔI_2_) before and after rebinding were examined. For this purpose, the MIP-based sensor prepared via EP was immersed in a 5 × 10^–11^ M IVM solution and tested at different durations (3, 5, 7, 10, 12, 15, and 20 min) using a thermoshaker (650 rpm, 25 °C). The results showed that ΔI_2_ reached its maximum value at 15 min. Therefore, a 15 min rebinding time was selected as the optimal condition for the MAA-IVM@MIP/GCE sensor, as illustrated in Figure S1E.

### 3.4 Analytical Performance of the MIP and NIP Sensors

The electrochemical properties and analytical capability of the MAA-IVM@MIP/GCE sensor were investigated using the [Fe­(CN)_6_]^3–^/^4–^ redox probe. DPV was applied as an indirect detection strategy for IVM under optimized conditions. In this method, instead of directly measuring the drug, changes in the probe’s electrochemical signal were monitored. When IVM molecules occupy the imprinted cavities on the MIP surface, electron transfer at the electrode interface is partially blocked, leading to a reduction in the redox peak current. This decrease, denoted as ΔI_2_, increases proportionally with the concentration of IVM.

A calibration curve constructed by plotting ΔI_2_ versus IVM concentration (1.0 × 10^–12^ – 1.0 × 10^–11^ M) exhibited excellent linearity **(** Figure 3A). The regression equation was ΔI_2_ (μA) = 4.54 × 10^12^ + 57.39, with a correlation coefficient (R) of 0.9918 (Table 1). From the slope of this curve and the standard deviation of the blank response, the limit of detection (LOD) and limit of quantification (LOQ) were calculated as 2.91 × 10^–13^ and 9.71 × 10^–13^ M, respectively.

2 LOD=3σ/slope

3 LOQ=10σ/slope

where σ = standard deviation.^29^

**Figure 3:**
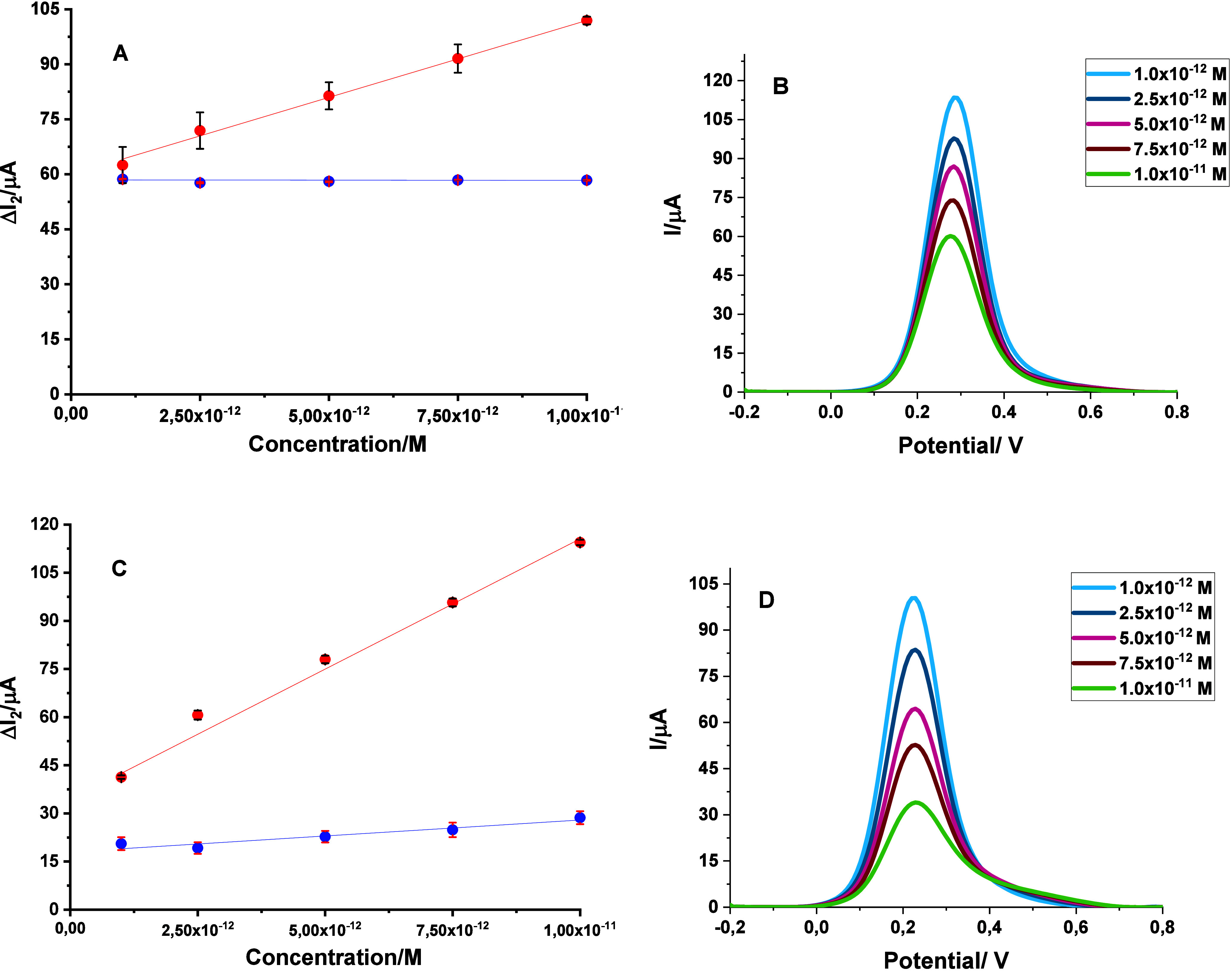
Calibration plots for IVM recorded with MIP- and NIP-based sensors in (A) standard solutions and (C) spiked serum samples, together with the corresponding DPV voltammograms illustrating sensor responses at different IVM concentrations in (B) standard solutions and (D) spiked serum. All measurements were carried out in 5 mM [Fe­(CN)_6_]^3–^/^4–^ using the DPV method.

**Table 1:** Regression Parameters for the Determination of IVM Using the MAA-IVM@MIP/GCE Sensor

	**Standard solution**	**Commercial serum sample**
**Linearity range (M)**	1.0 × 10^–12^ to 1.0 × 10^–11^	1.0 × 10^–12^ to 1.0 × 10^–11^
**Slope (μA M** ^**–1**^ **)**	4.54 × 10^12^	7.81 × 10^12^
**SE of slope**	1.79 × 10^11^	1.18 × 10^12^
**Intercept (μA)**	57.39	37.36
**SE of intercept**	1.68	7.22
**Correlation coefficient (R)**	0.9918	0.9900
**LOD (M)**	2.91 × 10^–13^	2.91 × 10^–13^
**LOQ (M)**	9.71 × 10^–13^	9.71 × 10^–13^
**Repeatability of response (RSD%)** ^a^	0.79	0.87
**Reproducibility of response (RSD%)** ^a^	1.93	1.67

a Each value is the mean of five experiments.

Further comparison between MIP- and NIP-modified electrodes confirmed the system’s selective recognition capability. The MIP electrode displayed an apparent, concentration-dependent suppression of ΔI_2_ (red curve). In contrast, the NIP electrode showed only negligible variations (blue curve), consistent with the absence of specific binding sites. These observations verify that the MAA-IVM@MIP/GCE sensor provides exceptional sensitivity and selectivity for ultratrace IVM detection.

### 3.5 Biological and Pharmaceutical Product Determination

The MAA-IVM@MIP/GCE sensor was successfully applied for the reliable detection of IVM in both pharmaceutical tablet formulations and spiked human serum samples. To assess its analytical performance, different concentrations of IVM (1.0 × 10^–12^ −1.0 × 10^–11^ M) were tested in serum. Within this range, the peak current responses exhibited a well-defined linear relationship (Figure 3C), expressed by the regression equation ΔI_2_ (μA) = 7.81 × 10^12^ + 37.36 with an excellent correlation coefficient (R = 0.9900). The sensor demonstrated remarkable sensitivity, with LOD and LOQ values of 2.91 × 10^–13^ M and 9.71 × 10^–13^ M, respectively, calculated from the regression data (Table 1).

Comparative calibration plots of MIP- and NIP-based electrodes in both standard solutions and spiked serum samples (Figures 3A and 3C) further confirmed the sensor’s selectivity. The MIP sensor exhibited a clear, concentration-dependent increase in ΔI_2_, whereas the NIP sensor response remained nearly unchanged. This contrast highlights the superior binding affinity and recognition capability of the imprinted electrode toward IVM. Figures 3A and 3B, respectively, display the resultant DPVs that were created by rebinding different concentrations of the linear curve in standard solution and serum samples.

To validate the sensor’s practical applicability, recovery experiments were performed (Table 2). The recovery values obtained for tablet and serum samples were 99.94% and 102.93%, respectively, confirming the high accuracy and reliability of the method for real sample analysis.

**Table 2:** Recovery Results of IVM Determined with the MAA-IVM@MIP/GCE Sensor in Tablet and Serum Samples

	**Pharmaceutical tablet form**	**Commercial serum sample**
**Label amount (mg)**	3.00	
**Found amount (mg)** ^a^	2.90	
**RSD%** ^a^	4.65	
**Bias%**	–3.20	
**Spiked amount (mg)**	10.00	10.00
**Found amount (mg)** ^a^	9.99	10.29
**Average recovery (%)**	99.94	102.93
**RSD% of recovery** ^a^	4.20	2.97
**Bias%**	–0.06	2.93

a Each value is the mean of five experiments.

### 3.6 Tap Water Recovery Study

To assess the sensor’s practical effectiveness and analytical robustness, recovery experiments were performed using tap water as an environmental test matrix. Assessing tap water illustrates the dependability of the sensor under actual environmental and ecological circumstances, thereby endorsing its viability for sustainable monitoring purposes. Recovery values ranging from 98.07% to 103.04% were obtained, demonstrating the accuracy and reliability of the proposed method for environmental analysis **(** Table 3 **)**.

**Table 3:** Recovery Results of IVM in Tap Water Samples with the MAA-IVM@MIP/GCE Sensor

**Sample Concentration (M)**	1 × 10^–12^	2.5 × 10^–12^	5 × 10^–12^
**Spiked Amount (M)**	6.5 × 10^–12^	5 × 10^–12^	2.5 × 10^–12^
**Found Amount (M)**	7.71 × 10^–12^	7.73 × 10^–12^	7.36 × 10^–12^
**Average recovery (%)** ^a^	102.80	103.04	98.07
**RSD% of recovery**	3.96	5.79	0.31
**Bias%**	2.80	3.04	–1.93

a Each value is the mean of five experiments.

### 3.7 Soil Extraction Recovery Study

To further evaluate the sensor’s performance in complex environmental systems, recovery experiments were carried out using soil samples. As a heterogeneous and demanding matrix, soil offers a realistic measure of the method’s resilience and selectivity. The recovery results showed that the sensor can accurately quantify the analyte even in solid samples with significant matrix interference. Overall, these findings confirm that the developed method is reliable, environmentally compatible, and effective for sustainable monitoring of contaminated soil environments **(** Table 4 **)**.

**Table 4:** Recovery Results of IVM in Soil Samples with the MAA-IVM@MIP/GCE Sensor

**Sample Concentration (M)**	1 × 10^–12^	2.5 × 10^–12^	5 × 10^–12^
**Spiked Amount (M)**	6.5 × 10^–12^	5 × 10^–12^	2.5 × 10^–12^
**Found Amount (M)**	7.47 × 10^–12^	7.41 × 10^–12^	7.24 × 10^–12^
**Average recovery (%)** ^a^	99.67	98.78	96.56
**RSD% of recovery**	3.70	1.67	1.35
**Bias%**	–0.33	–1.22	–3.44

a Each value is the mean of five experiments.

### 3.8 Imprinting Factor/Model Drugs

A primary aim of molecularly imprinted polymers is to achieve high selectivity for the target molecule, particularly in the presence of structurally similar analogs. To investigate this property, a selectivity study was conducted using competitor compounds with comparable chemical structures and properties.

In this study, the model drugs were selected based on their similarity in carbon number and molecular weight to IVM, since compounds with identical functional groups were not readily available. The MAA-IVM@MIP/GCE sensor’s selective binding to IVM was assessed against model drugs, including paclitaxel and ritonavir (Table 5). The results showed that the MIP sensor had a markedly higher affinity for IVM, demonstrating its excellent selectivity. This indicates that IVM interacts specifically with the recognition cavities formed during the imprinting process, which are precisely tailored to its size and shape. In contrast, the NIP sensor displayed a weak response, likely due to nonspecific interactions between the functional monomers and the model drugs, as it lacks the specific binding sites for IVM (Figure S2).

**Table 5:** Evaluation of the MAA-IVM@MIP/GCE Sensor’s Selectivity for IVM against Compounds of Comparable Size

	**MAA-IVM@MIP/GCE**		**NIP**		
	**ΔI**_**2**_ **/μA**	**k**_**(MIP)**_	**ΔI**_**2**_ **/μA**	**k**_**(NIP)**_	**k′**_**(MIP/NIP)**_
**Ivermectin**	81.39		58.06		
**Paclitaxel**	18.07	4.50	28.17	2.06	2.19
**Ritonavir**	8.96	9.09	12.95	4.48	2.03

### 3.9 Interference

IVM was tested at a concentration of 5 × 10^–12^ M in the presence of possible interfering species, including K^+^, NO_3_^–^, Na^+^, SO_4_^2–^, Mg^2+^, Cl^–^, dopamine (DOP), paracetamol (PAR), uric acid (UA), and ascorbic acid (AA). The results indicated that none of these interferents caused a significant change in the IVM peak current response. Recovery values ranged from 97.03% to 104.69% **(** Table 6 **)**. These findings confirm that the analytical efficiency of the MAA-IVM@MIP/GCE sensor remains stable even in the presence of interfering compounds (Figure S3).

**Table 6:** Influence of Various Interfering Substances on IVM Detection

**Interference**	**Recovery of IVM (%)**	**RSD (%)**
KNO_3_	100.11	3.89
MgCl_2_	104.69	1.13
Na_2_SO_4_	101.99	3.18
Dopamine	103.92	4.85
Paracetamol	101.58	1.08
Uric acid	97.03	3.93
Ascorbic acid	98.55	2.01

### 3.10 Binding Affinity and Isotherm Analysis

In order to assess the originality and efficacy of the proposed MIP sensor, an investigation into the binding affinity was conducted utilizing the Langmuir adsorption model. This model is particularly pertinent for MIP-based electrochemical sensors, as it presumes that the target analyte, IVM, attaches to a limited number of uniform, independent sites within the polymer matrix. The binding constant (K_a_, frequently denoted as K_L_) and the maximum binding capacity (*I*_max_) were ascertained by linearizing the Langmuir equation in the following manner:

4 $$\frac{1}{I}=\frac{1}{I_{\max}}+\frac{1}{I_{\max}K_{a}}\cdot \frac{1}{C}$$

5 $$I_{\max}=\frac{1}{b}$$

6 $$K_{a}=\frac{1}{m\cdot I_{\max}}$$

7 $$K_{d}=\frac{1}{K_{a}}$$

By plotting 1/I against 1/C, a linear regression was achieved (R^2^ ≈ 0.996) with a slope (m) of 6.39 × 10^–15^ and a Y-intercept (b) of 0.0103. The remarkable linearity confirms that the MIP–IVM interaction adheres to the Langmuir adsorption model. The saturation-type behavior observed experimentally (*I*_max_ ≈ 97.1) aligns with monolayer adsorption on uniform binding sites. The LOD for an electrochemical sensor is fundamentally associated with the binding constant (K_a_). A high K_a_ value (1.61 × 10^12^ M^–1^) signifies an extraordinarily strong affinity between the IVM molecules and the imprinted cavities. This robust chemical attraction guarantees that even at very low concentrations, the analyte is efficiently captured and retained at the electrode surface, leading to a sensitive signal response and a low LOD. Additionally, the dissociation constant (K_d_) was determined to be 6.2 × 10^–13^ M. This subpicomolar K_d_ value underscores the high thermodynamic stability of the MIP-IVM complex, further validating the sensor’s exceptional sensitivity and selectivity in comparison to traditional nonimprinted polymers.^30,31^

### 3.11 Comparison of Selected Analytical Methods

A thorough review of the literature revealed numerous effective strategies for IVM detection. While spectroscopic and chromatographic techniques deliver reliable results, they often require complex sample preparation, expensive instrumentation, and skilled personnel. By contrast, electrochemical methods are simpler and faster but sometimes lack sufficient selectivity for detecting drugs in biological samples. The sensor developed in this study overcomes these limitations, offering enhanced selectivity, rapid analysis, cost-effectiveness, and environmental friendliness. Application to real samples demonstrated that the MAA-IVM@MIP/GCE sensor provides accurate, fast, and user-friendly detection. A summary of various IVM determination methods is provided in Table 7.

**Table 7:** Comparative Overview of Analytical Techniques for IVM Determination

**Methodology**	**Linear range**	**LOD**	**Sample**	**Recovery**	**Ref**
**TLC**	1–50 ng/g	≤40 ng/g	cattle dung		^6^
**Amperometric flow-injection**	0.60–50 μmol/L	0.30 μmol/L	pharmaceutical drugs and urine samples	95–97%	^32^
**ESI LC-MS/MS**	1.0 −100.0 ng/mL	10 ng/mL	raw milk	85 to 105%	^33^
**LC-MS/MS**	1.5–500 μg/L	0.1 μg/kg	feces, soil, and sewage	92.27 ± 12.01%	^34^
**HPLC**	2 ppb to2 ppm	1 ppb	bovine feces	84%	^35^
**HPLC-fluorescence**	0.025–5 ng/mL	8.33 pg/mL	camel plasma samples	>70%	^36^
**ic-ELISA and lateral-flow immunochromatographic assay strip**	0.09–0.770 ng/mL	0.09 ng/mL	raw milk samples	94% to 112% and for the ICA strip from 110% to 125%.	^37^
**DPV**	9.99× 10^–7^ to 1.1 × 10^–4^ M	2.66 × 10^–6^ M	urine sample	100.953% to 105.2713	^38^
**MAA-IVM@MIP/GCE**	1 × 10^–12^ to 1 × 10^–11^ M	4.31 × 10^–12^ M	pharmaceutical drugs, tap water, soil	99.94% and 102.93%	This study

### 3.12 Computational Studies

DFT calculations were performed to determine the optimum template:monomer ratio and to elucidate possible template-monomer interactions. The structures of the template-monomer complexes at ratios ranging from 1:1 to 1:5 are presented in Figure 4.

**Figure 4:**
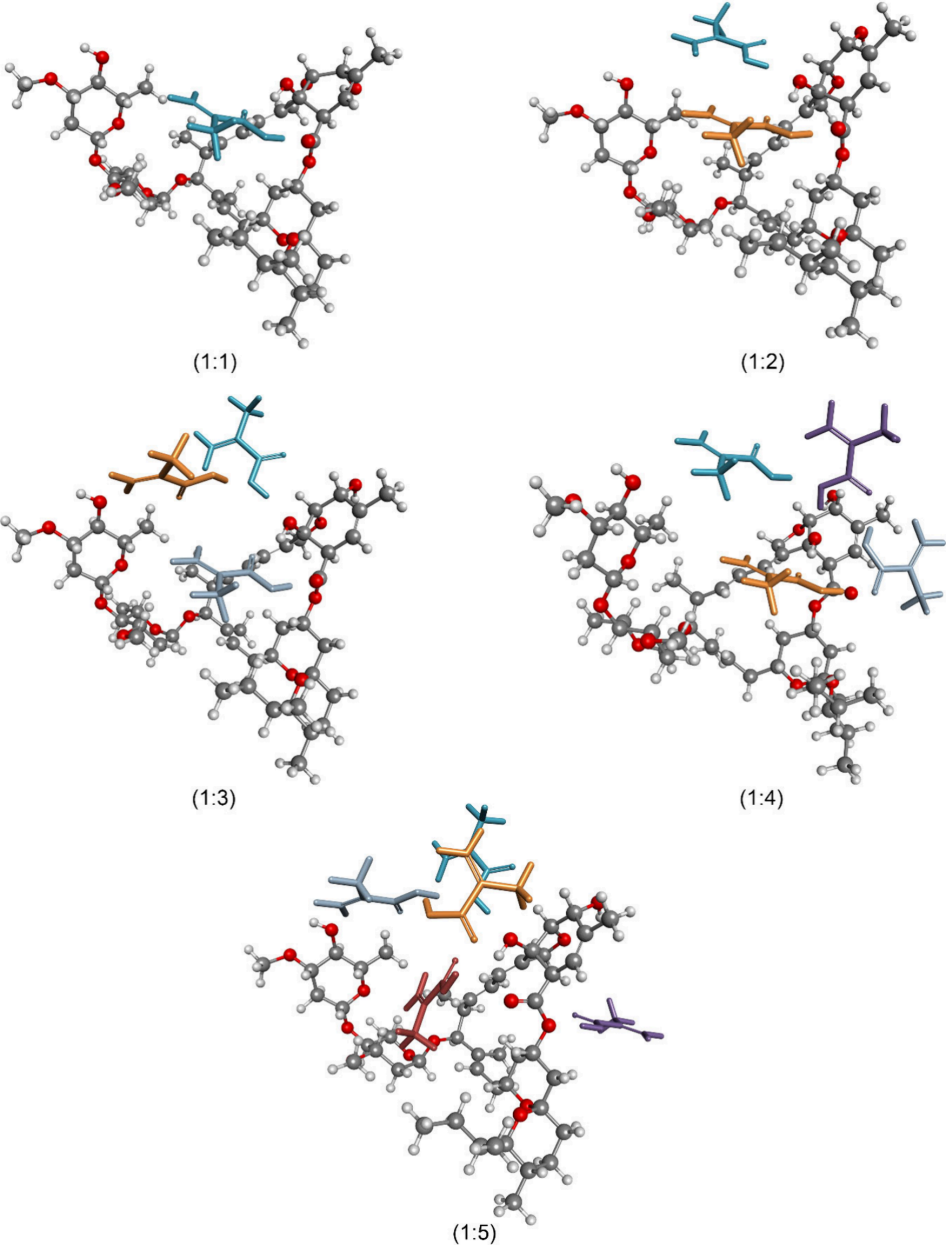
Geometry-optimized structures of the complexes at template:monomer ratios ranging from 1:1 to 1:5.

The binding energies of the complexes at ratios ranging from 1:1 to 1:5 were also calculated, and the results are summarized in Table S1. To investigate the effect of template:monomer ratio on binding affinity, both computational and experimental approaches were employed. The results reveal a nonlinear trend, with binding energy increasing up to the 1:3 ratio, plateauing at 1:4, and then decreasing at 1:5. The decline observed at 1:5 suggests that excessive monomer concentration may lead to nonspecific interactions or steric hindrance, reducing the effective binding strength.

In MIP-based sensor design, the strength of interaction between the template molecule and the polymer matrix is a key determinant of sensor performance. While sufficient binding strength is necessary to ensure selective molecular recognition, excessively strong interactions may hinder template removal and reduce the sensor’s reusability. Conversely, very weak binding may result in poor selectivity and low signal response. Therefore, the goal is not to achieve the highest possible binding energy, but rather to identify a balance that supports both recognition and regeneration.

Among the tested ratios, the 1:1 complex exhibited a binding energy of −57.12 kJ/mol, which reflects a moderate and well-defined interaction profile. This value is sufficiently strong to support selective recognition, while remaining low enough to allow efficient template removal and sensor regeneration. Importantly, experimental studies confirmed that the 1:1 ratio yielded the best sensor performance in terms of selectivity, signal intensity, and reproducibility. The agreement between computational predictions and experimental outcomes reinforces the validity of the 1:1 ratio as the optimal formulation for this system.

In addition, for the 1:1 template:monomer, which was also experimentally identified as the optimum ratio, the specific template-monomer interactions are illustrated in Figure S4. The results indicate that, in the template-monomer (1:1) complex, the template-monomer interactions are mediated through hydrogen bonding. In these interactions, the template molecule, ivermectin, acts as both a hydrogen bond donor and a hydrogen bond donor.

### 3.13 Green Profile of the Proposed Sensor

A sensor’s green profile highlights its commitment to environmental sustainability through eco-friendly materials, energy-efficient manufacturing, and minimal waste. This strategy aligns with green chemistry principles by conserving resources, reducing emissions, and using reusable parts to foster environmental responsibility.^19^ To evaluate the study’s green profile, three metrics were utilized: BAGI, AGREE, and AGREEMIP. The BAGI tool assesses the relevance of the developed method. The scale ranges from 25 to 60, with scores of 60 or above recommended. In this study, a score of 75 was obtained (Figure 5A). This result confirms that the method is effective.^39^ Furthermore, reusing the sensor reduces environmental impact and supports resource conservation, in line with GAC’s sustainability goals for sensitive and selective IVM detection. The AGREE metric evaluates the method against the 12 principles of GAC on a scale from 0 to 1, where higher scores indicate greater ″greenness.″ The investigation achieved a score of 0.64 with this tool (Figure 5B), highlighting advantages like reduced energy consumption and fewer hazardous chemicals. Nonetheless, the method has certain limitations, such as lacking in situ analysis, incomplete automation, and not accounting for renewable resources.^20^ For evaluating the synthesis of the Molecularly Imprinted Polymer (MIP), the AGREEMIP tool was employed. It considers energy needs, reagent environmental impact, and other synthesis factors across 12 criteria to generate a weighted score. In this study, the AGREEMIP score was 0.86, categorizing the sensor as “highly green” (Figure 5C). Therefore, AGREEMIP is a more effective GAC method than other approaches for specifically evaluating the environmental impact of MIP synthesis processes.^40^

**Figure 5:**
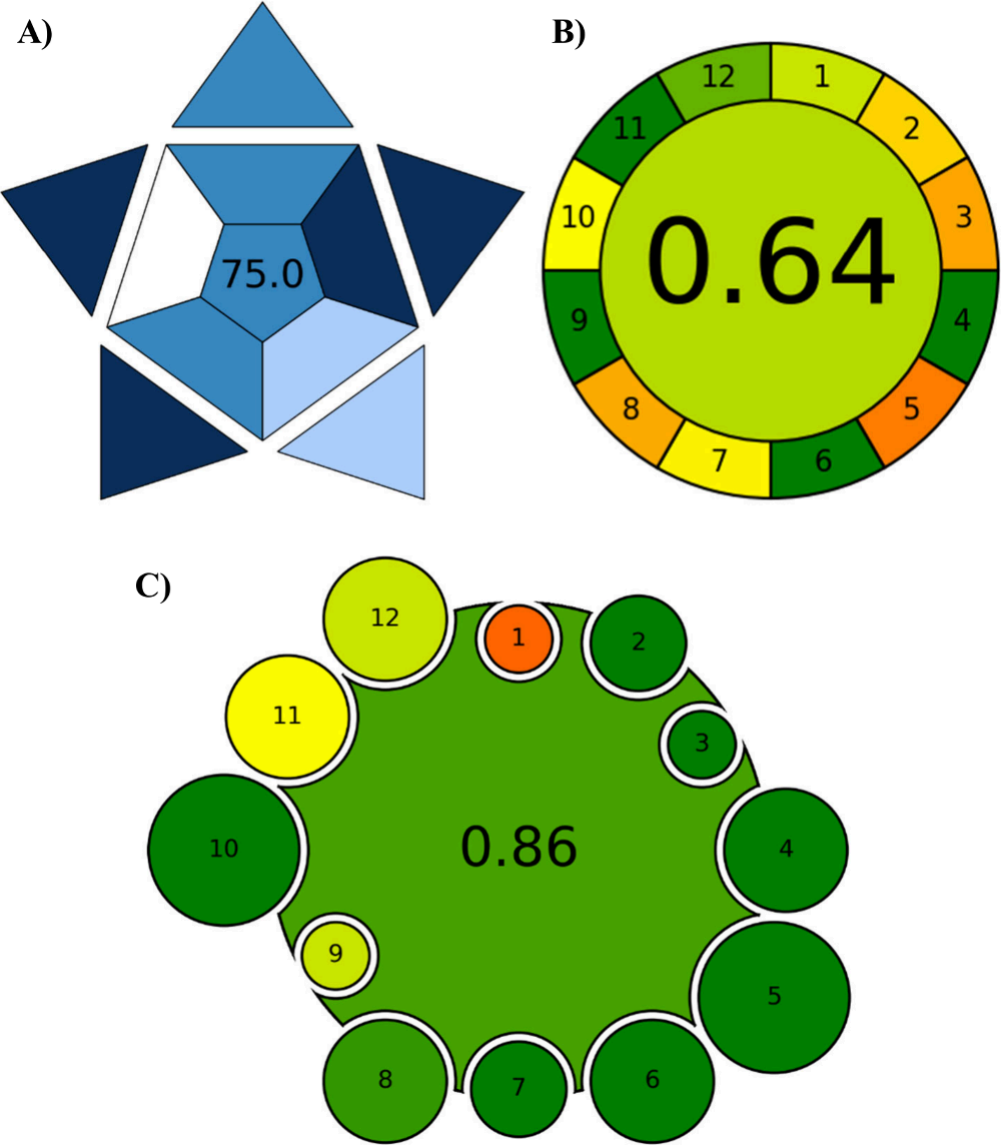
Pictograms for (A) BAGI, (B) AGREE, and (C) AGREEMIP.

## 4 Conclusions

This study’s electrochemical sensor represents a notable breakthrough in the field, employing molecular imprinting as an effective technique for precise chemical detection. The research primarily aimed to develop a MAA-IVM@MIP/GCE sensor using electropolymerization. The sensor demonstrated accurate recognition and binding to IVM molecules, offering notable benefits such as higher sensitivity and cost efficiency. Validation results, including recovery rates and RSD%, verified the sensor’s reliability and practical application. As the first MIP-based IVM sensor, the MAA-IVM@MIP/GCE system holds considerable promise for future applications, particularly in terms of portability, miniaturization, and rapid analysis. This research presents a versatile method for designing high-performance detection systems that integrate molecular imprinting with electrochemical techniques, effectively enabling accurate detection of IVM in real-world samples. Although the present study focused on steady-state quantitative analysis using DPV, the proposed sensor architecture is compatible with chronoamperometric or flow-based configurations, which could enable real-time monitoring in future developments. Ultimately, this study offers a novel approach to sensitive, rapid IVM detection and highlights the potential of MIP materials for developing future portable, point-of-care (POC), and lab-on-a-chip (LOC) diagnostic devices.

## Supplementary Material



## Notes

Supplementary figures and tables, including optimization parameters, selectivity and interference studies, and theoretical study results (PDF)
