# Supplementary material for: Green MIP-Based Electrochemical Sensing Platform for Environmental Ivermectin Analysis
Source: ACS Omega. 2026 Apr 30;11(18):27050–62. doi: 10.1021/acsomega.6c00641 (PMC13177245; doi:10.1021/acsomega.6c00641)
Supplement: Supplementary file 1 [file ao6c00641_si_001.pdf]

SUPPLEMENTARY MATERIALS

Green MIP-Based Electrochemical Sensing Platform for Environmental Ivermectin Analysis

Zeynep Aydemir[a], Beril S. Kaya[a,b], Setareh Dorreh[a], Abdullah Al Faysal[a,c], Taner Erdoğan[d] and Ayşegül Gölcü[a]*

[a]Istanbul Technical University, Faculty of Sciences and Letters, Department of Chemistry, Maslak, Istanbul, Türkiye.
[b]Istanbul Health and Technology University, Faculty of Pharmacy, Istanbul, Türkiye.
[c]Department of Chemistry, Faculty of Science, Atatürk University, Erzurum, 25240, Türkiye
[d]Kocaeli University, Kocaeli Vocational School, Department of Chemistry and Chemical Processing Technologies, Kocaeli, Türkiye.

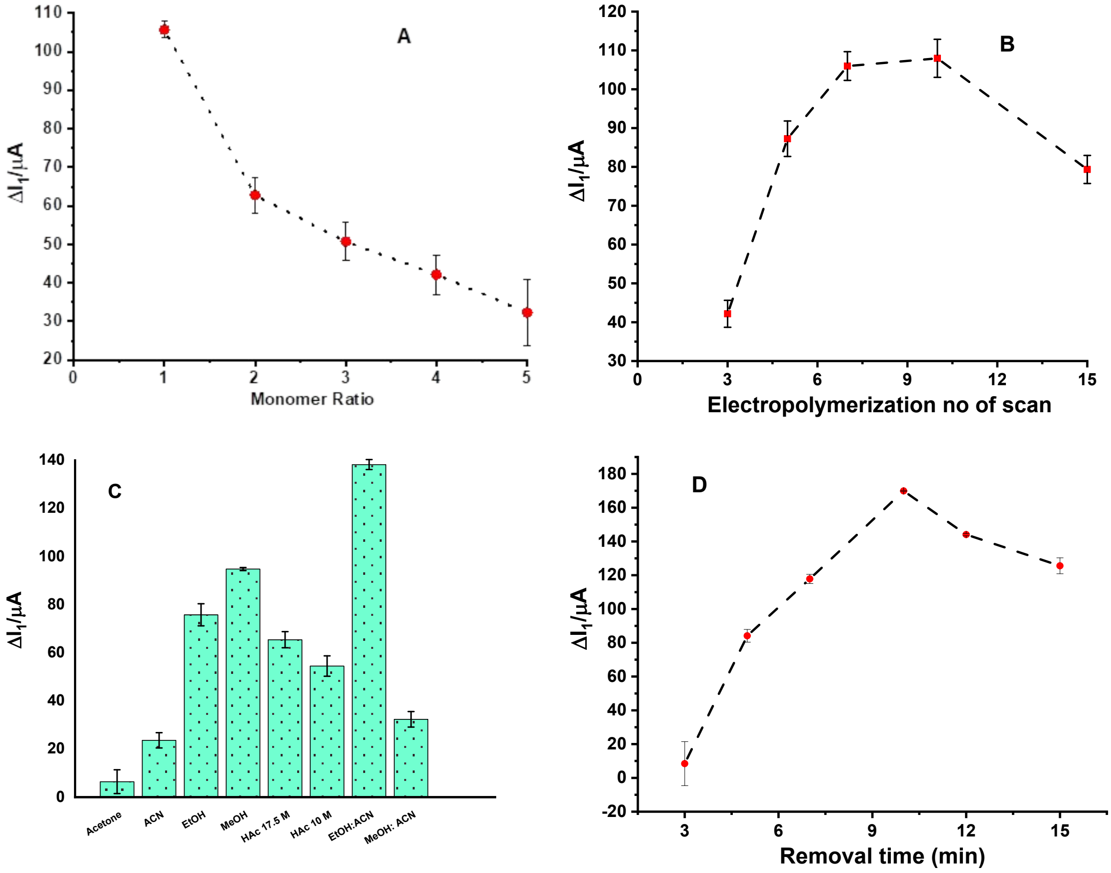

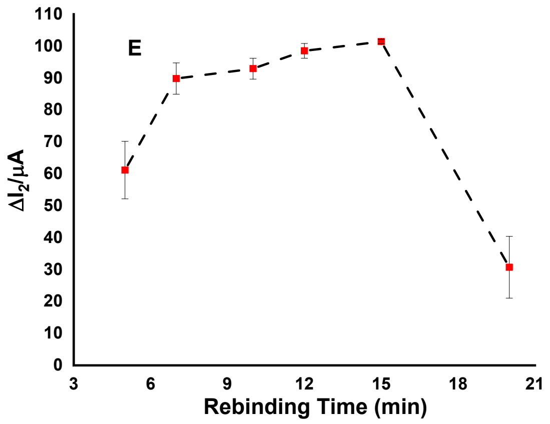

Figure S1. Variation of ΔI_1 values with respect to (A) monomer-to-template ratio, (B) electropolymerization cycle number, (C) different template removal solvents, and (D) removal time, along with ΔI_2 values as a function of (E) rebinding duration. All experiments were conducted using DPV in a $[Fe(CN)_6]^{3-/4-}$ redox probe solution.

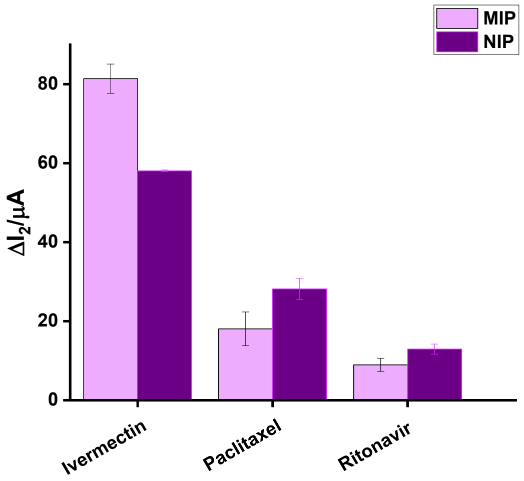

Figure S2. Selective response comparison of MAA-IVM@MIP/GCE and MAA-IVM@NIP/GCE sensors for IVM, paclitaxel, and ritonavir.

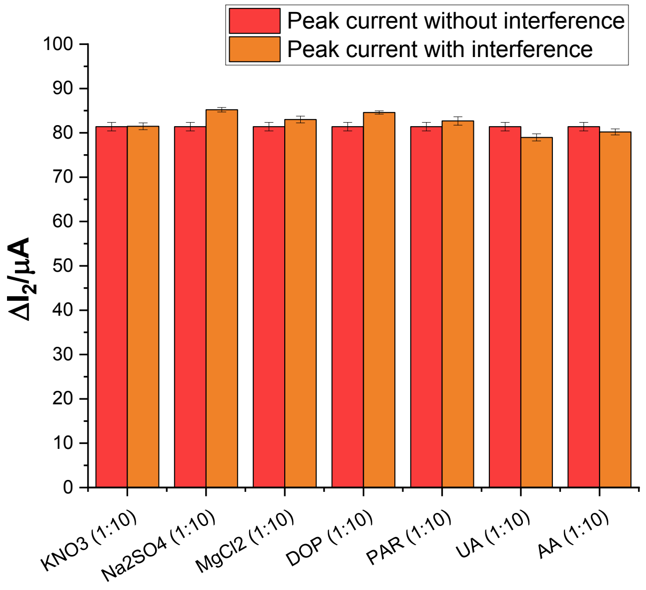

Figure S3. Response of the MAA-IVM@MIP/GCE sensor to 5×10^{-12} M IVM in the presence of potential interfering substances.

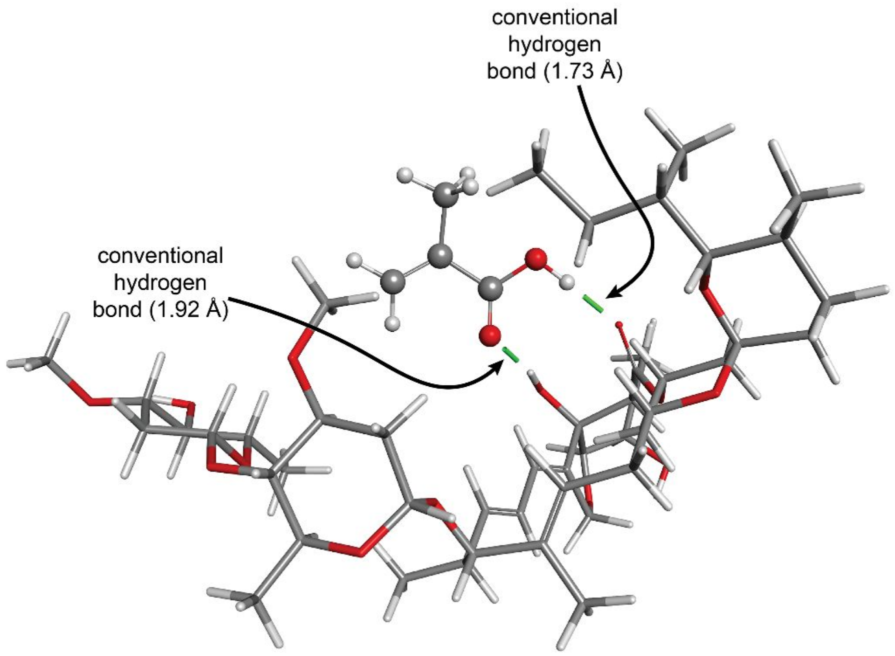

Figure S4. Template-monomer interactions in Ivermectin-MAA (1:1) complex.

Table S1. The calculated binding energies.

Ivermectin:MAA Ratio	Binding Energy (kJ/mol)
1:1	-57.12
1:2	-87.00
1:3	-101.43
1:4	-101.33
1:5	-87.46
